# Artificial intelligence in clinical genetics

**DOI:** 10.1038/s41431-024-01782-w

**Published:** 2025-01-13

**Authors:** Dat Duong, Benjamin D. Solomon

**Affiliations:** Medical Genetics Branch, National Human Genome Research Institute, National Institutes of Health, Bethesda, MD USA

**Keywords:** Data mining, Genetics, Genetics research

## Abstract

Artificial intelligence (AI) has been growing more powerful and accessible, and will increasingly impact many areas, including virtually all aspects of medicine and biomedical research. This review focuses on previous, current, and especially emerging applications of AI in clinical genetics. Topics covered include a brief explanation of different general categories of AI, including machine learning, deep learning, and generative AI. After introductory explanations and examples, the review discusses AI in clinical genetics in three main categories: clinical diagnostics; management and therapeutics; clinical support. The review concludes with short, medium, and long-term predictions about the ways that AI may affect the field of clinical genetics. Overall, while the precise speed at which AI will continue to change clinical genetics is unclear, as are the overall ramifications for patients, families, clinicians, researchers, and others, it is likely that AI will result in dramatic evolution in clinical genetics. It will be important for all those involved in clinical genetics to prepare accordingly in order to minimize the risks and maximize benefits related to the use of AI in the field.

## Introduction

The entire field of medicine is being impacted by artificial intelligence (AI). Though AI is now a ubiquitous (and often overhyped) presence in the lay and scientific press, this ongoing transformation of medicine will not happen overnight, and will advance with fits and starts. It is nevertheless probable that AI will significantly change the practice of medicine in a relatively short amount of time. Predicting exactly *how* medicine, including clinical genetics, will evolve due to AI is difficult due to many regulatory, legal, financial, logistic, ethical, computational, and other factors, and the fact that many different stakeholders disagree about optimal approaches and outcomes. As an upshot, while medicine will change dramatically due to AI, exactly *what* the future of medicine holds – and how these changes will affect patients, clinicians, researchers, and society – remains murkier.

With these unsettling questions in mind, this review article seeks to do two main things. First, we will review aspects of AI to help familiarize readers with basic concepts, especially in the context of clinical genetics. Second, we will briefly review previous, current, and emerging applications of AI in clinical genetics. While this review is not meant to be systematic or comprehensive (and will focus on the most recent “game-changing” AI developments), it may nonetheless be useful to readers who, like all of us, are constantly bombarded with information about AI, but who may feel less knowledgeable about the underlying methods, applications, and considerations. At the same time, this review will touch on some challenging and controversial questions regarding AI in clinical genetics. As a side note, readers may well disagree with many of the points and opinions in this review article. This is a good thing, as there are many looming questions, and the “right answers” remain unclear. In other words, vigorous debate and engagement are important, and for this to happen, readers should endeavor to understand and grapple with the topic.

Though many practitioners may not be aware, aspects of clinical genetics already depend heavily on AI. For example, when a clinician sends a specimen to a lab for genetic testing, responds to a patient’s email, or searches clinical information via Google (or, of course, uses a large language model (LLM) like ChatGPT, Claude, or Gemini), they are leveraging different forms of AI – often, they may use several different types of AI simultaneously. However, the degree to which clinical genetics will depend on AI in the future will increase substantially. As an analogy, we currently take it for granted that much of medicine depends on using computers in many ways, shapes, and forms – after clinicians, the computer is arguably more integral to medicine than any other entity. In the future, AI will be as inextricably linked to medicine as computers are today. The huge – and uncomfortable and even downright scary – difference is that, unlike computers, AI may soon supplant many human functions in medicine. This does not necessarily mean that humans will leave medicine, but future human roles may be very different.

## Aspects of clinical genetics and AI

Clinical genetics is but one of many medical fields. Among these fields, and for several intertwined reasons, clinical genetics may be especially poised to be impacted by AI [1]. First, there are insufficient clinical geneticists and other genetics experts [2, 3]. Second, there are thousands of known genetic conditions, many of which are rare. Even if there were a surfeit of expert geneticists, no single clinician can be knowledgeable about all (or even most) genetic disorders [4]. Third, genetic conditions are often severe and multisystemic, causing significant personal, familial, and societal impact; early diagnosis can be important for maximally beneficial, cost-effective care [5–8]. Despite advances in sequencing and related technologies and the known benefits of timely diagnosis, patients often experience years-long diagnostic delays and lack of access to state-of-the-art testing – this is especially problematic in historically disadvantaged populations and less wealthy geographic regions [9–11]. Because of these clinical genetics attributes, AI may help fill gaps to efficiently and accurately diagnose, investigate, manage, and communicate with patients and families [1, 2, 12]. That is, leveraging AI may enable non-geneticist clinicians as well as patients to compensate for the lack of direct access to geneticists.

Relatedly, clinical genetics has always been (perhaps uniquely among medical fields) focused on discovery. Many dozens of novel causes of genetic conditions are reported annually [13, 14]. AI applications can support such research while also helping meet the pressing clinical needs outlined above. For example, a recent paper studied large cohorts of patients who had undergone exome sequencing, and reported 28 “new disease genes” involved in neurodevelopmental disorders [15]. As part of the gene prioritization strategy, these researchers used a method of variant analysis called CADD (Combined Annotation Dependent Depletion), which leverages a type of machine learning (ML) algorithm called support vector machines (see the next section for an overview of AI types) [16]. CADD is now used routinely by clinical labs and researchers worldwide, though newer, deep learning (DL)-based methods are starting to supplant ML approaches considered cutting-edge a short decade ago [17]. Interestingly, AI may be especially valuable in finding explanations for currently unexplained conditions that have more occult or complex causes. As discussed further in the sections below, beyond identifying the causes of genetic disorders, AI will also be integral to identifying new therapeutics to treat genetic conditions.

However, many of the same aspects of clinical genetics that make it suited for AI applications also result in challenges. The rarity of individual genetic conditions means that the amount of data available to train AI models is often not ideal despite long-term, dedicated efforts towards compiling highly useful databases of conditions, genetic variants, and so on (e.g., OMIM, Orphanet, ClinGen, ClinVar, gnomAD, etc.). Further, datasets of affected populations do not equally represent all affected populations, meaning that the performance will vary across different groups of patients [18]. Additionally, while the causes of many genetic conditions are known, there are many nosologic questions, such that it remains unclear how conditions should be analytically “lumped or split” [4, 19, 20]. In summary, when studying and applying AI, clinicians and researchers must keep in mind nuances specific to clinical genetics [21, 22]. In order to ensure that AI models and tools work well in clinical genetics, efforts will be required related to activities such as collecting and annotating datasets, implementing and finetuning models, and generally carefully checking to see how applications work in different populations [23, 24].

## Basic concepts and definitions

Before we delve further into AI in clinical genetics, we will review some general terms and concepts. The following section may be of historical and general interest to some, though the descriptions are intentionally high-level and do not account for subtleties and exceptions.

**Fig. 1:**
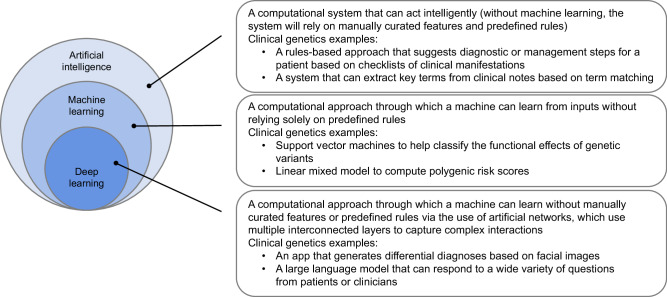
Relationships, general definitions, and examples of artificial intelligence, machine learning, and deep learning.

First, AI is not a monolith – there are many subcategories and different methods within the field (see Fig. 1 for general definitions and examples). Although some terms used to describe AI are intuitive, it is useful to explain what they mean and how they relate to each other. “Artificial intelligence” refers to a computational (non-organic) system that can act in ways that “seem smart.” This AI system may be able to accomplish a specific, narrow task very well, such as identifying certain types of genetic changes from data produced by a genomic sequencer or suggesting the most likely type of genetic cardiomyopathy based on echocardiogram images; this might be called “narrow AI”. On the other hand, an AI system that can perform a very broad range of tasks with human-like intelligence would be termed “general AI”. There are naturally disagreements about these definitions, including when true “general AI” will be achieved.

Many philosophers and cognitive scientists also argue about whether such AI systems are *actually* intelligent, or just seem intelligent. This connects to other conundrums, such as the nature of consciousness (of which there is shockingly little understanding). But let us set aside fascinating questions such as these and focus on the pragmatic fact that AI systems can do clever things and accomplish complex tasks. This definition of AI connects to one of the original ideas in the field of AI, the Turing test, described in 1950 [25]. Named after the British genius and polymath Alan Turing, a computer was said to pass the Turing test (which was initially referred to as the “imitation game”) if it could act intelligently enough to seem convincingly human. Once the realm of science fiction, we now take such abilities for granted due to the widespread availability of LLMs – in fact, general-purpose LLMs already exceed most humans across many tasks. As a sad aside, but to further emphasize human shortcomings, Turing’s life is a stark reminder that human intelligence should not be equated with human compassion or ethical wisdom. After his key role in helping defeat Germany in World War II, Turing was forced to undergo chemical castration when his homosexuality came to light. He either committed suicide or died due to accidental cyanide poisoning shortly thereafter.

That ghastly lesson notwithstanding, it is again important to underscore there are many forms of AI – the term “AI” does not refer to one single entity. In fact, for decades after Turing wrote his seminal paper, rules-based systems were the leading type of AI. This refers to AI that uses a set of rules to dictate the actions of an AI system. For example, programmers might build a pre-LLM chatbot-type system that could provide suggestions for genetic testing or management based on responses to checkboxes where a user would fill in a patient’s clinical features. However, there are limitations to the types of complexities that such a system could handle. Leading AI models are no longer based on rules-based systems due to challenges capturing all possibilities. However, these rules-based approaches may still be combined in very useful ways with the more modern AI methods described below.

Within the broad field of AI, the next subcategory is machine learning (ML). Again, the term roughly explains what is meant: a computer (machine) that can learn and improve without relying solely on explicitly programmed rules. However, the ML model would still largely depend on manually curated inputs (such as specific clinical variables when analyzing a cohort of patients, as described below). There are several different types of ML, including supervised learning, unsupervised learning, and reinforcement learning. To help explain this, we will use genetics examples.

Let us imagine that we are analyzing a cohort of patients with Mendelian forms of diabetes mellitus, like Maturity Onset Diabetes of the Young (MODY, MIM #606391). For each person, we know (based on genetic testing) which condition they have, and we also have a great deal of clinical data. From this information, we can select a subset of useful input features (these would be the “manually curated inputs” referred to above), such as glucose and insulin values, exocrine pancreatic biomarkers, renal imaging and related lab values, and so on. We want our supervised ML model to be help us figure out which individuals are most likely to have which conditions based just on clinical data. In supervised learning, we would label the individuals according to their underlying genetic conditions, such as the various MODY types. We would feed these data into the ML model – this labeled data would help the model differentiate individuals with specific genetic conditions. While we trained the ML model with data on individuals with known diagnoses, this supervised approach could be useful because we could eventually use it on individuals for whom a diagnosis is not yet known – the model could help ensure clinicians suggest appropriate initial testing, management, and so on.

Now, let’s take an unsupervised ML model, and again apply it to our cohort of people with diabetes mellitus. In this unsupervised ML approach, we would not label the individuals as having specific conditions such as forms of MODY; there would be no “ground truth”. We would see how, based on differences in the clinical data, the unsupervised ML might separate or categorize these individuals into distinct clusters. Perhaps, without even being explicitly told to do so, it might helpfully separate people in a way that would suggest common biologic (genetic) causes in different clusters, which could be investigated. There are also semi-supervised ML approaches, which combine labeled and unlabeled data.

Finally, reinforcement learning is another type of ML approach, where the objective is to identify a set of actions that help maximize a specified “reward”. Reinforcement learning can be helpful in situations involving sets of actions that change over time. For example, let’s imagine a different ML analysis, again related to MODY. A researcher may wish to predict how dietary, sleep, and other daily habits affect laboratory values, like glucose. The objective is to find the optimal daily habits that are most beneficial to the patient (e.g., in this case, the pattern that offers the best glucose control). A person’s habits may change over time – initially, they may sleep 10 hours (h) a night, then they may for a time only sleep an average of 6 h, then may later settle on sleeping about 8 h per night. Reinforcement learning could help estimate to what extent different choices over time affect a person’s lab values – the model would then be able to recommend the best set of actions for an individual. An everyday analogy for how reinforcement learning works is the way that a person trying to get cellphone coverage physically moves around – by seeing where they get the best signal, they might learn that a certain location works best to accomplish their goal (in this case, to have a clear phone call) [26].

One of the ways that researchers suspect that AI models may become increasingly intelligent will involve designing and training themselves using forms of reinforcement learning. This may involve computational approaches that are not necessarily intuitive to humans, but which may be very powerful, similar to how an AI system beat the professional Go player Lee Sedol using a brilliant but unconventional move [27].

One type of ML that has gained great attention of late is deep learning (DL). DL uses a structure called artificial neural networks (ANNs). “Artificial” again refers to the fact that there is no human or other organic brain involved. “Neural networks” refers to the way that data pass through interconnected layers of the DL model, which is inspired by the way that neurons connect in the brain. The reason that this type of learning is called “deep” is because there are many layers; these layers consist of a layer for data input, a layer for data output, and multiple “hidden layers” between the input and output layers. In general, the fact that there are many layers allows DL models to be trained to do complex, specialized tasks that can be useful in clinical genetics (as well as for many other purposes). For example, DL is often used in computer vision, where a computer analyzes images. In clinical genetics, DL may be used to “classify” (categorize) facial images to see what genetic condition a person may have based on their appearance [8, 28]. In general, to accomplish this, the DL model will be trained on many facial images, including images of people with different genetic conditions. At first, the model learns to identify simple things, like the edges of the face versus the background. More layers allow increased complexity, such as the ability to detect different parts of the face (e.g., the mouth, nose, and eyes). Then, the model can be trained to detect different genetic conditions based on the shapes of the facial features. It could even be trained to detect subsets of a condition, such as which gene might be most likely to be involved in a person with Noonan syndrome [29].

The advent of DL is one of the primary reasons that AI is so prevalent now, and so poised to impact clinical genetics, medicine overall, and society. Other central reasons for AI’s rise include increased computational power, especially as relates to special types of computer chips called graphic processing units (GPUs). GPUs were originally used to render complex video game graphics but are now central to AI due to their capacity to perform specific mathematical operations in parallel. To provide a sense of scale, OpenAI used 25,000 NVIDIA A100 GPUs (“NVIDIA A100” refers to the GPU type) to train its GPT-4 model. This required ~100 days of constant training, with an estimated cost of ~$100 million USD. Another reason for the rise of AI is the growth of the internet, which enabled large datasets – including words, images, audio, and video – to be amassed, annotated, and made available to train AI systems [30]. These datasets can be directly and indirectly used for many AI training purposes. For example, models used to identify genetic changes by assessing the faces of people with genetic conditions were built on top of (through something called “transfer learning”) models that leverage vast general databases of facial images [23].

Lastly, powerful and popular “generative AI” methods involve AI that is used to make (generate) something, such as words, still or video images, sounds, or some combination of these. Generative AI involves different types of deep learning. One important breakthrough in DL involved the use of transformers, a type of DL architecture (“architecture” refers to the structure of the DL framework). Transformers incorporate a self-attention mechanism – this allows models to capture contextual relationships, such as how different parts of a sentence relate to each other [31]. This breakthrough was instrumental in LLM development, though the way the term “LLM” is often used now is something of a misnomer, since what many refer to as LLMs are currently multimodal – they can input and output different forms of data, not just text. Generative AI can be used in many ways in clinical genetics, such as to study how certain variables affect the diagnosis of genetic conditions. For example, researchers used generative AI methods to change the facial expressions of people with genetic conditions (such as ones associated with smiling expressions, like Angelman and Williams syndrome) to study how models as well as humans use these types of clue to recognize genetic conditions [32]. Understanding how these and many other variables affect AI models will be important as AI is increasingly implemented in the clinic.

## AI in diagnosis

In clinical genetics, diagnosis often relies on a combination of clinical (including physical examination, laboratory-based, radiologic, and other observable manifestations) and molecular findings. AI can be used to support virtually all aspects of the diagnostic process; that is, AI models can be used to assess many different data types about a patient with a suspected genetic condition to help arrive at a potential diagnosis.

One major way that AI is increasingly used in clinical genetics involves assessing physical examination-based features; this ties back to computer vision. A huge leap forward in medical computer vision was a consequence of breakthroughs in DL. A DL model, called “AlexNet” after the name of one of the inventors, was used to win the (nonmedical) Image Net competition in 2012 (Geoffrey Hinton, the senior author on the AlexNet paper, received the 2024 Nobel Prize in physics for his DL contributions) [33]. In the several years that followed, DL models using the type of approach pioneered by AlexNet showed impressive results in a number of clinical areas where images are important, such as in pathology, ophthalmology, and radiology [34–36]. In addition to matching or outperforming humans at these types of clinical tasks, such as assessing medical images to determine what illness a person might have, AI systems are able to detect things that humans cannot, such as sex or anemia via retinal images [37, 38].

Specific to clinical genetics, computer vision leapt visibly onto the scene in 2010s, long before AI was a household word. One notable medical example was the app called “Face2Gene”, which introduced many clinical geneticists to DL methods. This product involves the use of DL to provide differential diagnoses based on facial images (later, other phenotypic data could be input as well). Interestingly, part of the reason for the rapid adoption of Face2Gene may have involved its ease-of-use; like ChatGPT more than a decade later, the fact that Face2Gene had an intuitive interface that made it widely accessible may have been integral to its extensive use. As one demonstration of its impact, in a recent scoping review about DL in clinical genetics, 58 of 134 (43%) articles (published from January, 2015 – June, 2021) in this topic area involved the use of Face2Gene. AI-based tools like Face2Gene may be especially helpful in providing access to clinical genetics expertise in parts of the world lacking in in-person access to such expertise [39]. One advantage of these methods is that AI approaches like “one-shot” or “few-shot” learning can be applied to identify conditions that, due to their rarity, are not well-represented in databases used to train AI [40]. This is an example of a way in which AI methods can be adjusted in the field clinical genetics so that they may work for even conditions that are so rare that they are not well-represented in training databases.

In addition to analyzing facial images, these types of DL-based computer vision approaches have been applied to many other types of images encountered in clinical genetics, including images of skin lesions, ophthalmologic studies, microscopy data, radiologic images, ECGs, EEGs, and other image types [8, 18, 22, 28, 41, 42]. In the spirit of Face2Gene, there are several publications and applications that use different image types to provide genetic differential diagnoses, such as “Eye2Gene” and “Bone2Gene” (https://eye2gene.com/; https://bone2gene.org/), though such endeavors are still primarily in the research phase [42]. There are also increasing efforts from international collaborations to combine different data types (e.g., photographs, radiologic images, clinical information, etc.) into consolidated databases for both research and clinical purposes [43]. Due to these types of efforts, is likely that future clinicians will not need to turn to different tools to analyze different types of images (along with other data) – unified tools will be able to process most relevant types of data. In addition to identifying individuals as having known genetic conditions, these tools may also be useful to help provide explanations for currently undiagnosable patients: AI can be used to group or analyze individuals for whom genomic testing has not yet revealed answers [8].

While DL-driven computer vision caused huge waves in the medical world starting in the 2010s, LLMs and other forms of generative AI exploded onto the scene about 10 years later. In clinical genetics, AI techniques involving the analysis of text using natural language processing (NLP) have been employed for many years, but LLMs (often combined with more traditional NLP methods) appear to offer much broader and more powerful abilities to analyze text along with other data types. Several studies soon after the launch of ChatGPT in 2022 highlighted abilities relevant to clinical genetics, such as identifying genetic conditions from clinical descriptions and responding to clinical questions [44–47]. While these studies showed variable performance of LLMs, the rate of improvement of LLMs in the last several years underlines their enormous potential.

For example, in a recent randomized controlled trial, ChatGPT outperformed physicians in diagnosing general medical conditions based on standardized vignettes [48]. Most notably, in this study, ChatGPT also outperformed physicians that were allowed to use ChatGPT, questioning the idea that “AI will not replace clinicians, but clinicians who use AI will replace those who do not.” Such emerging studies point to a future where physicians may indeed be replaced by AI at certain tasks (especially involving diagnosis), while other “physical” activities, such as performing surgeries and delivering babies, will be relatively preserved, at least for the near term. For example, in clinical genetics, a patient diagnosed by a geneticist as having Lynch syndrome may undergo frequent colonoscopies in order to detect and therefore treat neoplasms early. While a gastroenterologist may perform the colonoscopy, an AI model may soon conduct the primary analysis of the colonoscopy images and biopsy results, write the report, and later respond to follow-up questions from the patient.

Finally, AI has already had profound impacts in genetic testing laboratories, and AI’s impact will accelerate. Clinical genetics has always been a technology-focused field, primarily due to the centrality of genetic testing. In the last several decades, these technologies have changed dramatically, progressing from stages where cutting edge genetic testing involved (to name a few, and in rough chronological order): karyotype; single gene sequencing; microarray; next-generation sequencing (which enabled large gene panels, exome, and genome sequencing); long-read sequencing; and clinical multi-omics [49]. Each of these testing types is amenable to AI-based analysis, though the specific type of approach depends on the assay [50]. Currently, clinical genetic testing may employ AI-based support to perform tasks such as extracting and correlating phenotypic and genotypic data, identifying and assessing variants of interest, and communicating and explaining testing processes and results [17, 51–55].

AI’s recent rise coincided closely with the need in laboratory-based clinical genetics for more efficient approaches to handle testing volumes efficiently and cost-effectively. This need was driven in part by increasing use of genetic testing in many parts of the world, including because of the challenges involved in handling bioinformatic analyses manually. That is, since large sequencing panels, exomes, and genomes generate much more data than single-gene studies, and as more individuals were undergoing clinical genetic testing, it was impossible to adequately scale the workforce to handle clinically-informed genetic analyses through approaches that were largely manual. Simultaneously, in regions such as the United States, financial pressures (i.e., increased pressure on sequencing companies to become profitable, which occurred for multifactorial reasons, including due to interest rate changes) meant that labs were forced to aggressively reduce costs. As AI tools were coincidentally much more useful and accessible, laboratories aggressively pursued such methods. One has only to peruse recent advertisements by clinical genetic laboratories to see how central AI is becoming to laboratory processes.

## AI in management and therapeutics

AI may ultimately replace many of the diagnostic activities that today occupy the bulk of clinical genetics practice. This will likely force clinicians who work in clinical genetics to shift to taking a larger role in active patient management and treatment. With biochemical genetics as a notable exception, clinical genetics has traditionally focused more on diagnosis than treatment. While clinicians may no longer be as important in the diagnostic space, there may be room for the shift to management due to progress in areas such as gene therapy, gene editing, antisense oligonucleotide, and other “molecular” approaches to directly treating people with genetic conditions [1, 56]. AI will play an increasingly central role in this area as well [1]. As the vast majority of genetic conditions still lack direct treatments, there is a pressing need to accelerate knowledge about the underlying biologic causes (which can in turn unlock therapeutic possibilities) as well as to find novel ways to treat genetic conditions [53, 57]. AI is already impacting biological discovery in ways that may lead directly to therapeutics in clinical genetics.

Many readers will be familiar with the stupendous success of AlphaFold, which used DL methods to predict protein structure (garnering the 2024 Nobel prize in chemistry), and which can be directly relevant to identifying possible treatments [53]. As a more recent example, researchers recently analyzed the “dark proteome” and revealed that there are possibly thousands of previously overlooked genes. This multi-pronged endeavor involved several AI-based approaches and revealed potentially druggable targets that are already being investigated [58]. As another example, researchers have used generative AI approaches to help create new systems that can be used for gene editing [59, 60]. These types of studies show how research in general will increasingly shift in the near-term (see “The future” section below for further discussion on this topic) – powerful AI tools will be ubiquitously used alongside clinical and wet-bench approaches to enable new ways to develop therapeutics for genetic conditions.

Apart from AI’s role in identifying new therapeutics, there are other ways that AI will affect patient management. In the traditional clinical genetics model, a person suspected of being at risk of having a genetic condition is typically first seen by a clinician such as a pediatrician or general practitioner. Clinical signs of a genetic condition would lead to a referral to a geneticist. This genetics or other specialist would help diagnose the patient, usually through some type of genetic testing. Then, tailored management considerations, often performed by an appropriate subspecialist like an oncologist or cardiologist, would be implemented based on the specific diagnosis. For example, if a patient were identified as having Von Hippel Lindau syndrome based on family history and subsequent genetic testing, cancer surveillance by an oncologist would be indicated. A person found to have vascular Ehlers-Danlos syndrome would be counseled to avoid dangerous activities, and have specific care related to pregnancy [4]. In this context, AI is already increasingly used as a source for management information. For example, OpenEvidence (https://www.openevidence.com/), a medical LLM, can offer general management information (though not specific medical advice about a particular patient at the time of writing); importantly, this source includes specific links and resources, which helps with LLMs issues like hallucinations and verifiability [46].

In addition to augmenting traditional diagnosis and management practices, including via “precision medicine” approaches described above, AI may help enable genetics to have a broader clinical reach. For example, AI-supported tools may help comb electronic medical record (EMR) and other systems to identify individuals, based on clinical features, who are likely to have a genetic condition; previous analyses have shown that such individuals are often overlooked [61]. Similar approaches can be used in a genotype-first approach, where AI-supported interrogations can help identify individuals within large cohorts who may be at risk of adverse outcomes, and who therefore could benefit from clinical diagnosis and management [62]. To be clear, these approaches may not require sophisticated AI methods, but it is likely that AI will be increasingly used to detect and therefore treat potentially impacted individuals, including to help analyze data patterns that suggest a person may be at risk due to a combination of genotypic and phenotypic findings.

## AI in clinical support

In an ideal world, clinical geneticists would spend the vast majority of their time diagnosing, managing, studying, and otherwise supporting patients and families affected by genetic conditions. In reality, administrative tasks impart an enormous burden and greatly reduce the time that clinicians can devote to patient care. Although caution should be taken with generalizations, one survey in a US state estimated that less than one-quarter of genetic counselors’ time was spent in face-to-face patient care; other reviews of the clinical geneticist workforce in high-income countries depict an extremely heavy administrative load [63, 64].

AI may provide much-needed help with administrative burdens that reduce the ability to provide direct patient care (and which contribute to clinician burnout and job dissatisfaction). For example, AI may help write clinical notes, respond to emails, renew prescriptions, complete genetic testing, and deal with paperwork requested by payors. As with other areas, this type of AI support started before the LLM tsunami, such as through the use of chatbots to respond to patient queries about laboratory testing [55]. Several recent papers highlight the potential impact of LLMs in this area; these studies also raise the specter of human replacement by AI, especially as AI tools become more agentic (act more autonomously). For example, a study compared physician and LLM responses to general medical questions from an internet forum. The LLM answers were felt to be of overall higher quality and were rated as more empathetic than physician responses [65]. LLMs have also been shown to be potentially helpful in many other areas (including areas that often involve specific complexity in clinical genetics due to the complicated and esoteric nature of the field), such as documenting and simplifying informed consent documents [66, 67].

These AI applications engender understandable excitement. However, in many healthcare systems, these type of AI interventions may be a double-edged sword, as the availability of AI may be used as rationale to remove resources, increase patient volumes, decrease clinician numbers, and so on. Further, the use of AI to help with administrative tasks, such as leveraging LLMs for documentation of medical notes and other information in electronic medical records (EMRs), should not be viewed as an immediate panacea, and may, at least in the near-term, actually cause problems if not handled carefully [68].

## The future

AI will affect the field of clinical genetics profoundly. To conclude this review, we would like to extend the discussion to offer several succinct predictions. These may well be wrong, though the degree to which they are wrong will only be revealed with time – these are provided to be intentionally provocative and hopefully lead to useful discussions that may ready our field for change. We outline three main areas below, though there are certainly alternative schema, and these areas overlap considerably such that they should best not be viewed as discrete entities. In the discussion below, “short-term” refers to ~1-2 years, medium-term refers to ~2–5 years, and longer-term refers to changes that will take at least 5 years.

First, there are the effects of AI on clinicians. In the short term, the degree to which AI affects clinical genetics will be highly variable – this pattern is very similar to what we have seen with the adoption of genomics. Some clinicians and systems will embrace the use of AI, and view it as a competitive advantage, especially in fragmented healthcare systems. Others, either due to understandable concerns about AI, or due to lack of resources and training, may initially shy away from AI. In the short and medium-term, AI is likely to be very useful for administrative tasks, such as clinical documentation and managing onerous paperwork. In the longer-term, as clinical genetics is currently a largely diagnostic field, and as AI-based chatbots and other tools become increasingly better at both diagnosis and communication, there is a significant chance that genetics clinicians will need to pivot to skills that cannot be easily performed via AI. Such skills include in-person conversations with patients and families – for those patients and families who still prefer this – and “physical”, procedure-based medical activities that are less easily replaced by AI (at least for now, pending further breakthroughs in robotics).

Second, there are laboratorian geneticists, including those who primarily analyze, interpret, and report genomic data for clinical purposes. This group might be the first impacted by large-scale replacement by AI, though individual laboratories may titrate the small percentage of cases that require human intervention, with some labs much more AI-automated than others. In the medium-term, tests like clinical exome and genome sequencing will be performed similarly to the way in which non-invasive prenatal testing (NIPT) is handled now, where the vast majority of tests are resulted with minimal individual human oversight, while a few outliers are tagged for human review. In the slightly longer-term, doing things like analyzing a genome will become similar to how a complete blood count (CBC) or basic chemistry panel is performed now. Naturally, there will be new, more complex tests, such as combining epigenetic, transcriptomic, proteomic, and other data, but these will, sooner or later, also fall under the spell of automation.

Finally, there is research related to clinical genetics. In the short term, AI will be used as a powerful tool to enable increasingly impactful breakthroughs, including about the causes, mechanisms, and treatments of genetic diseases. In the medium-term, though some feel this will take longer, it is likely that foundational models will become available that are both more intelligent and more strategic – across all human subjects – than even the most brilliant principal investigators. At that point, the model of research may flip entirely, such that AI systems plan, analyze, and report research, perhaps directing and using humans to do tasks such as collecting samples. Though this prediction may feel threatening (or downright dystopian), a rosier view is that such changes could have tremendous benefit for clinical genetics as well as the rest of human health. Overall, while these predictions may turn out to be quite wrong, we have high confidence that AI will significantly change clinical genetics. It would be prudent to prepare accordingly.
